# High temperature corrosion and oxide scale formation of nickel in molten NaOH at various basicity levels

**DOI:** 10.1016/j.heliyon.2024.e31995

**Published:** 2024-05-31

**Authors:** Birgitte Stoffersen, Daniel John Cooper, Morten Stendahl Jellesen, John Hald

**Affiliations:** a Seaborg Technologies, Titangade 11, 2200, Copenhagen, Denmark; b Section of Materials and Surface engineering, DTU Construct, Technical University of Denmark, 2800, Lyngby, Denmark

**Keywords:** Molten salt, High temperature corrosion, Sodium hydroxide

## Abstract

The corrosion behavior of alloy Ni 201 in molten sodium hydroxide (NaOH) at 600 °C was investigated at varying basicity levels of the molten NaOH. The ability for Ni 201 to form passivating oxides was investigated after immersion tests varying from 70 to 340 h under atmospheres of argon and argon with different partial pressure of water. Morphology and thicknesses of the corrosion products were characterized by Scanning Electron Microscopy (SEM) and crystallography of the corrosion products by X-ray Diffraction (XRD). Dynamic polarizations were made to investigate the effects of basicity and electrochemical potential.

The results showed that Ni 201 corroded at a reduced rate in molten acidic NaOH compared to neutral NaOH due to the formation of NiO. The oxide scales formed on Ni 201 in acidic NaOH were shown to grow non-parabolically and did not result in full corrosion protection as the oxide scales showed crack development over time.

## 1 Introduction

In recent years, extensive efforts have been made developing Generation IV nuclear reactors, aiming at producing clean, safe, and cost-efficient energy to meet the increasing energy demand in a sustainable manner, while being resistant to proliferation. One of the promising reactor technologies in Generation IV nuclear reactors are molten salt reactors (MSR), which are most commonly reactors making use of fissile material being dissolved in a circulating fuel salt. Fused sodium hydroxide is of interest as moderator in MSRs because it possesses the ability to slow down neutrons, offers stability towards irradiation [1], and is considered cost-effective being abundantly available. However, molten sodium hydroxide is generally severely corrosive at high temperatures [[2], [3], [4]].

Previous efforts selecting structural materials that will not undergo extensive degradation in contact with sodium hydroxide at high temperatures have been reported in literature. Generally, nickel-based alloys are reported as having the highest resistance to corrosion in fused NaOH in static conditions [[3], [4], [5]].

To extend Pourbaix's methodology of estimating regimes of immunity, passivation, and corrosion to cover molten salts, a relationship similar to pH in aqueous solutions defining the acid-base properties of molten salts has been established [6].

In molten hydroxides, the OH^−^ anion is amphoteric. The OH^−^ anion can accept a proton and become H_2_O, which acts as an acid and donate a proton and become O^2−^, which acts as a base. Molten hydroxides dissociate per equation (1) [6].

(1) $$2{OH}^{-}⇌\;H_{2}O+O^{2-}$$

The equilibrium constant of the dissociation reaction is given in equation 2.

(2) $$K=a\left(H_{2}O\right)\times a\left(O^{2-}\right)$$

where a(H_2_O) is the activity of H_2_O and a(O^2−^) the activity of O^2−^ and K is the equilibrium constant. The activities in equation (2) can be replaced with concentrations (equation (3)) [7].

(3) $$K=c\left(H_{2}O\right)\times c\left(O^{2-}\right)$$

A logarithmic value of the equilibrium constant K is defined in equation 4.

(4) pK=−logK

The acidity and basicity of the of the hydroxide melt can be defined as the negative logarithm of the concentration of H_2_O and O^2−^ respectively, as given in equations (5), (6)

(5) $$pH_{2}O=-\log\;c\left(H_{2}O\right)$$

(6) $$pO^{-2}=-\log\;c\left(O^{-2}\right)$$

which implies that pK can be defined as in equation 7.

(7) $$pK=p\;\left(H_{2}O\right)+p\left(O^{-2}\right)$$

In molten NaOH, the basicity is defined by the dissociation of NaOH into H_2_O, which is defined as an acid and Na_2_O, which is defined as a base [8]. And as such the basicity of molten NaOH can be controlled by the concentration of H_2_O and Na_2_O [5,6,[9], [10], [11]]. A NaOH melt can be considered acidic when the concentration of H_2_O is greater than the concentration of Na_2_O and basic when the concentration of Na_2_O is higher than that of H_2_O. Accordingly, molten sodium hydroxide saturated with H_2_O or Na_2_O are considered the limits for acidity and basicity range respectively [5,6,10,12,13]. A completely anhydrous NaOH can be considered a neutral melt and can be obtained by heating and dehydration under dry argon atmosphere [13].

Several studies have investigated the corrosion behavior of nickel in molten hydroxides. Experiments conducted under dry argon atmospheres have consistently shown formation of nickel oxide (NiO) on the surface of the nickel substrate [2,3,14]. The NiO formation has been proposed to proceed according to equation (8) [15].

(8) $$Ni+2NaOH=NiO+{Na}_{2}O+H_{2}$$

Goret & Tremillon studied the electrochemical behavior of metals in molten NaOH–KOH mixture at 227 °C by cyclic voltammetry. The study showed that the corrosion products forming on Ni in molten NaOH–KOH is dependent on the acid-base properties of the melt. Ni in acidic NaOH–KOH (hydrated) forms NiO as per equations (8), (9)), being insoluble in the acidic melt, however NiO can be further oxidized to NiO_2_ as per equation (10) at higher potentials.

(9) $${Ni}^{2+}+{2OH}^{-}=NiO+H_{2}O$$

(10) $${NiO+2OH}^{-}=NiO_{2}+H_{2}O+2e^{-}$$

In basic NaOH (enriched with O^2−^), Ni forms NiO_2_^2−^ as per equation (11), which is soluble in the melt. At high basicity NiO is unstable and dissolves to form NiO_2_^2−^ as stated in equation (12). At higher potentials NiO_2_ forms as per equation (13).

(11) $$Ni+2O^{2-}-2e^{-}=NiO_{2}^{2-}$$

(12) $$NiO+O^{2-}=NiO_{2}^{2-}$$

(13) $$NiO_{2}^{2-}-2e^{-}=NiO_{2}$$

This study investigated the influence of the basicity level of molten NaOH at 600 °C on the corrosion behavior of nickel. Experiments were carried out in neutral NaOH and two acidic conditions.

## 2 Experimental

### 2.1 Material

Immersion tests were carried out using coupons (30 x 20 × 3 mm) with a Ø 6 mm hole. The chemical composition provided by the manufacturer *Special Metals* is given in Table 1. The coupons were ground mechanically to a 0.2 Ra finish.

**Table 1:** Chemical composition of Ni 201 coupons as stated by the manufacturer.

Element	C	Mn	Fe	S	Si	Cu	Ni	Ti	Mg
Wt. %	0.02	0.26	0.02	0.0002	0.06	0.24	99.1	0.05	0.003

Dynamic polarization was carried out using samples of Ni 201 wire with a diameter of 2 mm in as received condition. The composition given by the supplier is given in Table 2.

**Table 2:** Chemical composition of Ni 201 wire as stated by the manufacturer.

Element	C	Mn	Fe	S	Si	Cu	Ni	Ti	Mg
Wt. %	0.01	0.035	0.03	0.001	0.041	0.012	99.85	0	0.02

### 2.2 Immersion tests

**Fig. 1:**
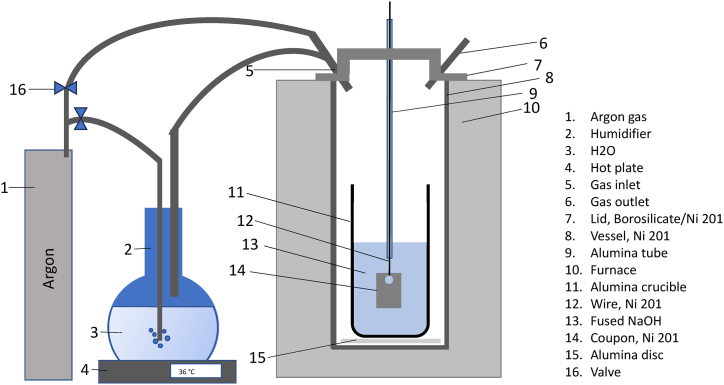
Illustration of experimental test setup for immersion and dynamic polarization. The dynamic polarization was made with additional counter and reference electrode and the working electrode was replaced with a wire sample.

Ni 201 coupons were exposed to NaOH at 600 °C for 70–340 h in a cover gas of argon atmosphere and argon atmosphere with varying partial pressures of water, corresponding to different levels of basicity of the melt, given in Table 3. The acidic conditions were achieved by passing of agon gas through a water bath before the argon was introduced into the experimental setup. Rahmel & Krüger [16] investigated the solubility of water in molten NaOH, which showed that the behavior of water vapor follows Henry's Law stating a direct correlation between the quantity of water within the melt and the partial pressure of water vapor up to temperatures of 500 °C. In this work, it is assumed that the concentration of water in the melt increases with the increasing partial pressure of water vapor above the melt. The partial pressure of water was applied by bubbling argon through a water bath held at 36 °C and 60 °C corresponding to partial H_2_O pressures of 0.059 atm and 0.197 atm respectively [17]. For experiments in neutral NaOH, where the cover gas consisted of dry argon, the water bath was bypassed, and the argon flowed directly from the argon supply to the inlet of the experiment (Fig. 1). All experiments were carried out in triplicate to verify replicability of the results.

**Table 3:** Nomenclature of the immersion tests.

Atmospheres	Nomenclature
• Dry argon • Argon with a H_2_O partial pressure = 0.059 atm • Argon with a H_2_O partial pressure = 0.197 atm	Ar/t Ar/p_H2O_(0.059)/t Ar/p_H2O_(0.197)/t

t = Exposure time, 70, 170 or 340 h.

An alumina crucible containing 500 g NaOH of 99.5 % purity with impurities of Na_2_CO_3_ was placed in a sealed vessel made of Ni 201, Fig. 1. Ni 201 coupons were hanging on a 2 mm thick Ni 201 wire through a hole in the coupons. The salt was heated in steps to dehydrate it. During heating and cooling of the NaOH the coupons were positioned above the melt under supply of dry argon cover gas with a flowrate of 100 ml/min. When the temperature reached 600 °C, the coupons were immersed into the molten NaOH and the cover gas was changed to the atmosphere required for the experiment. For the experiments under Ar/pH_2_O(0.059) condition the argon cover gas was directed through the water bath held at 36 °C, for Ar/pH_2_O(0.197) condition, the argon was led through a water bath held at 60 °C, whereas for the experiments carried out in cover gas of dry argon, the water bath was bypassed.

Post immersion, the coupons were rinsed from residual salt deposits and fragmented corrosion products by sonication in demineralized water for 10 min. Detached corrosion products were collected for X-Ray diffraction analysis.

### 2.3 Dynamic polarization

Dynamic polarization was carried out using the same setup as the immersion tests, Fig. 1. For the polarization, the lid of the vessel was a metal lid of Ni 201 as they were less prone to breakage than the lids of borosilicate. The tests were carried out under atmospheres of argon, argon with a partial water pressure of 0.059 atm and under argon with a partial water pressure of 0.197 atm. The working electrode (WE) was 2 mm thick Ni 201 wire, the reference electrode (RE) was Na/Na+ and the counter electrode (CE) was Ni201. The Na/Na + electrode was produced before each test in a glovebox. The reference electrode consisted of an α-alumina tube with a closed β-alumina bottom. 0.2 g of sodium was placed in the bottom of the β-alumina tube and a molybdenum wire was added. The tube was carefully sealed with silicone sealant inside the glovebox. Such type of reference electrode has been described to be stable in molten salt [18].

An alumina crucible was filled with 465g of NaOH and heated to 600 °C. When the temperature reached 600 °C the molten NaOH dwelled for 3 h at the test condition (Ar, Ar/pH_2_O(0.059) or Ar/pH_2_O(0.197)) before the measurements were started. The measurements were carried out with a Biologic VMP-300 multichannel potentiostat. The open circuit voltage (OCV) was measured for 20 min before the potential was swept from −0.5 V to 1.0 V vs OCV with a scan rate of 10 mV/s. The electrode was immersed 30 mm, which corresponds to a surface area of 1.95 cm^2^.

### 2.4 Characterization

A Quanta FEG 250 Analytical ESEM was used to analyze thickness and morphology of the corrosion products. Oxide thicknesses were evaluated by cross sectional analysis of three replicate coupons by mounting the coupons in epoxy and stepwise polishing with SiC paper to grit 4000, followed by diamond polishing solution for final polishing.

X-ray diffraction patterns of the coupon surfaces and corrosion products were generated using Bruker 08 Advance with a Co Kα X-Ray source with a step size of 0.03° and a scan time of 6 s. Analysis of detached corrosion products was made by collecting the fragmented corrosion products on carbon tape.

## 3 Results and interpretation

### 3.1 Immersion tests

Post exposure the coupons were sonicated in demineralized water for 10 min where a large quantity of corrosion products spalled off coupons of Ar/170 compared to coupons of Ar/p_H2O_(0.059)/170 and Ar/p_H2O_(0.197)/170. Weight losses in percentage of the original coupons after cleaning are listed in Table 4. The weight loss is significantly higher in neutral NaOH compared to acidic NaOH.

**Table 4:** Weight losses in percentage of the original weight after 170 h exposure to NaOH at 600 °C at various basicity levels.

Experiment	Weight loss of the original coupon (%)
Ar/170 Ar/p_H2O_(0.059)/170 Ar/p_H2O_(0.197)/170	5.9 ± 1.9 % 0.6 ± 0.1 % 0.1 ± 0.1 %

**Fig. 2:**
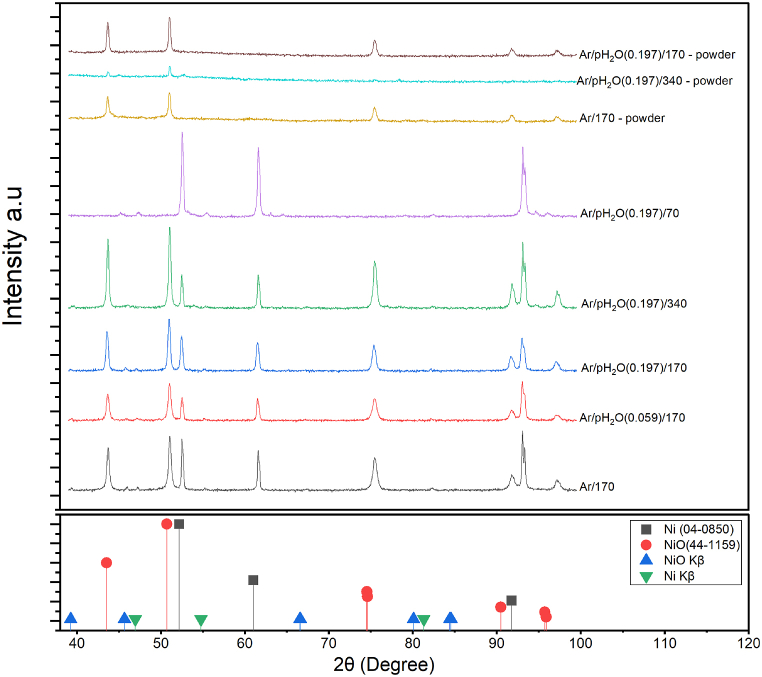
X-ray diffractogram of Ni 201 coupons and detached corrosion products (powder) post exposure to NaOH at 600 °C at various durations and basicity levels.

Fig. 2 shows the X-ray diffraction data of the cleaned immersion test coupons and corrosion product powders. NiO is the only corrosion product detected after immersion test of all three experimental conditions. The coupons that were exposed to NaOH under Ar/pH_2_O(0.197) show increasing intensity of the NiO peaks with increasing exposure time, which indicates growth of the oxide scale. The corrosion product that spalled off post exposure was also analyzed with XRD and only NiO was detected.

**Fig. 3:**
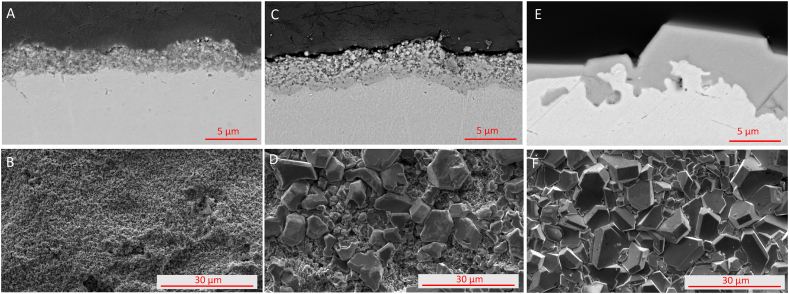
Micrographs of cross section and surface morphology of Ni 201 after 170 h of exposure to NaOH at 600 °C under varying basicity. A + B) Ar/170 C + D), Ar/pH_2_0(0.059)/170, E + F)) Ar/pH_2_O(0.197)/170.

Fig. 3 shows the morphology of the outer surface of exposed coupons in cross sectional and top view. Ar/170 exhibits a layer of oxide with a porous structure. Ar/p_H2O_(0.059)/170 has a layer of oxide with a duplex morphology, i.e. having a compact inner layer and an outer porous layer. Ar/p_H2O_(0.197)/170 has the most compact layer of oxide on the surface with a surface morphology that consists of a homogeneous and non-porous granular structure.

**Fig. 4:**
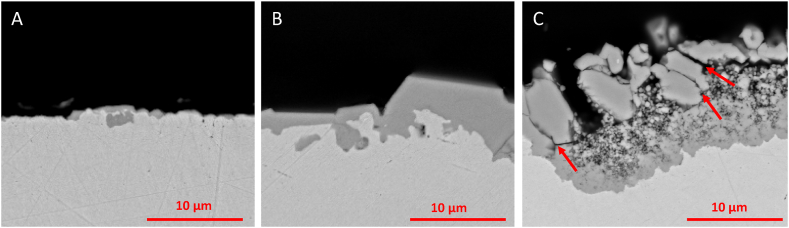
Cross sections of Ar/pH_2_O(0.197) after A) 70 h, B) 170 h, C) 340 h.

Fig. 4 shows the cross sections of coupons exposed to NaOH at 600 °C under atmospheres of argon with a partial pressure of water of 0.197 atm after exposure for 70, 170 and 340 h. The thickness of the oxide scale increased with time. The coupons exposed for 340 h show a duplex structure with a fine-scale porous inner layer and a larger granular outer oxide with substantial cracks marked by red arrows.

**Fig. 5:**
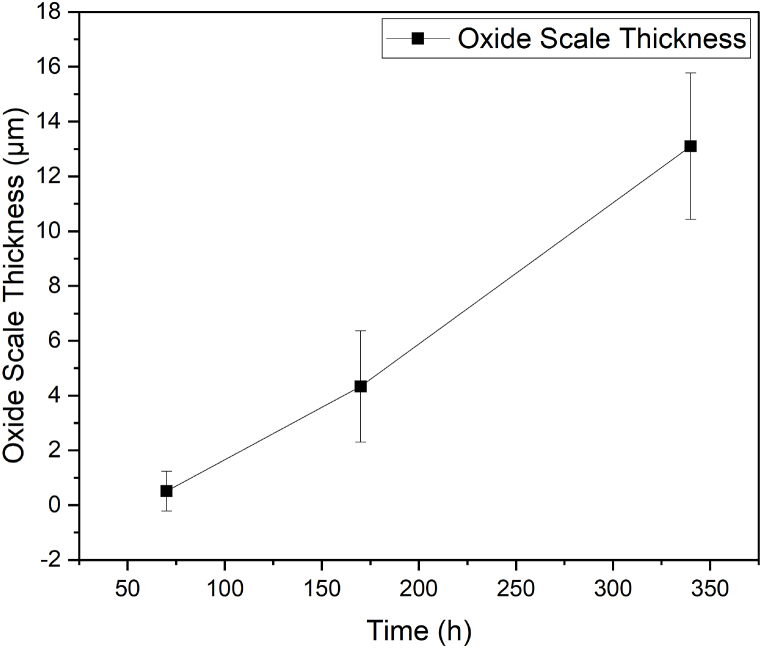
Oxide scale thickness (μm) as a function of time (h) for Ni 201 immersed in NaOH at 600 °C with a partial pressure of water of 0.197 atm.

Fig. 5: Oxide scale thickness (μm) as a function of time (h) for Ni 201 immersed in NaOH at 600 °C with a partial pressure of water of 0.197 atm Fig. 5 shows a plot of oxide thickness as a function of time on the coupons exposed to NaOH at 600 °C under atmospheres of argon with a partial pressure of water of 0.197 atm for different durations. Each data point is an average of the oxide thickness measured by cross sectioning in nine different locations of each of the triplicated coupons. The graph shows that the growth of oxide on Ni 201 is non-parabolic. Table 5 shows the weight losses of the coupons exposed to NaOH at 600 °C under atmospheres of argon with a partial pressure of water of 0.197 atm for different durations. The decrease in weight loss from 70 h to 170 h of exposure may be explained by the increase in oxide scale thickness. From 170 h to 340 h of exposure, the weight losses were increased by more than an order of magnitude.

**Table 5:** Weight losses in percentage of the original weight after 70, 170 and 340 h in acidic NaOH at 600 °C.

Experiment	Weight loss of the original coupon (%)
Ar/p_H2O_(0.197)/70 Ar/p_H2O_(0.197)/170 Ar/p_H2O_(0.197)/340	0.20 ± 0.09 % 0.11 ± 0.05 % 1.13 ± 0.02 %

### 3.2 Dynamic polarization

**Fig. 6:**
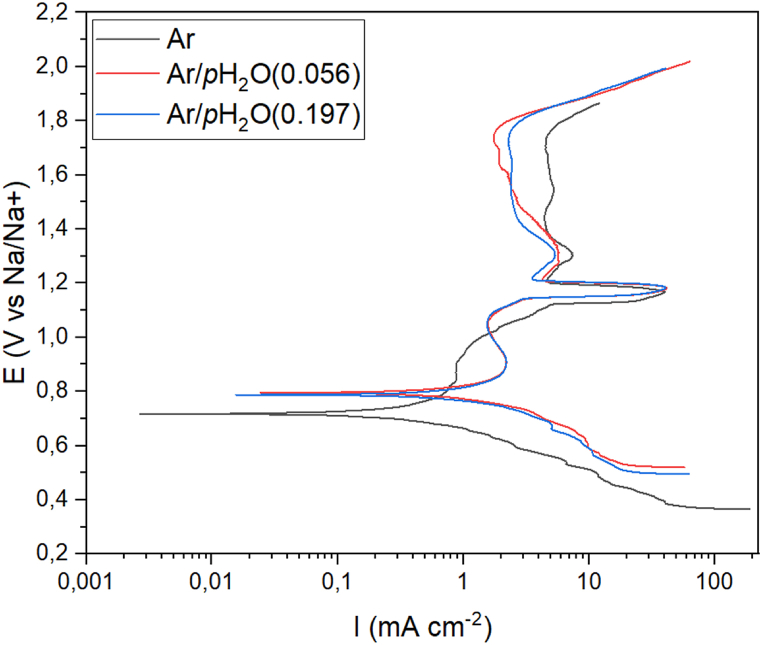
Polarization curves of Ni 201 in NaOH at 600 °C under various basicity levels.

Fig. 6 shows the polarization curves for Ni 201 in NaOH at 600 °C under various basicity levels. Ecorr was 0.718 V vs Na/Na + for the samples exposed to NaOH at 600 °C under atmosphere of argon and 0.793 V and 0.785 V vs Na/Na + for samples exposed under an atmosphere of argon with a partial pressure of water of 0.059 atm and 0.197 atm respectively. The decreased corrosion potential for Ni 201 in neutral NaOH indicates that Ni 201 acts less noble in neutral NaOH compared to in acidic NaOH. The cathodic region of the polarization curves shows higher current density levels for the coupons exposed to acidic NaOH compared to neutral NaOH. The anodic regions of the polarization curves are similar for samples exposed to cover gas of argon with partial pressures of water of 0.059 atm and 0.197 atm with a passive region from 0.85 V vs Na/Na + until a current increase at 1.15 V vs Na/Na+ (A). After the large current increase at 1.15 V vs Na/Na+ the current decreases again and is followed by a smaller current increase at 1.3 V vs Na/Na+ (B), whereafter the current density decreases and remains at a stable value of approximately 0.4 mA/cm2 until 1.8 V vs Na/Na+. The anodic region of the polarization curve for samples exposed to neutral NaOH showed less passivation behavior up to 1 V vs Na/Na + whereafter the current density increases around 1.15 V vs Na/Na+ (A) similar to samples in acidic conditions. After the large current increase at 1.15 V vs Na/Na+, the curve for neutral NaOH follows the same trend as measurements in acidic NaOH reaching stability at 1.4 V vs Na/Na + until 1.8 V vs. Na/Na + where current density again increases.

Oxidation of Ni to NiO according to equation (8) takes place at potentials above the corrosion potential and until the large current increase at 1.15 V vs Na/Na+. The current increase at 1.15 V (A) is expected to be due to formation of NiO22- according to equation (12) [6,9] and the smaller current increase at 1.3 V (B) caused by the formation of NiO2 according to equation (10). The current increase at 1.8 V vs Na/Na+ is ascribed to the formation of oxygen gas as per equation (14) [19].

(14) $$2{\text{OH}}^{-}-2e^{-}=0.5\;O_{2\;}\left(g\right)+H_{2}O$$

## 4 Discussion

The extent of corrosion of Ni 201 in molten NaOH at 600 °C depends on oxide formation, the passivating properties of the oxide, and thus the morphology of the oxide scale. In this study, Ni 201 showed oxide formation in both neutral and acidic NaOH after immersion. However, with a denser oxide scale formed in NaOH under cover gas of argon with a partial pressure of water of 0.197 atm.

Based on the increase in material loss and the lower corrosion potential for coupons subjected to neutral NaOH compared to acidic NaOH, it can be inferred that Ni 201 exhibits reduced corrosion resistance in neutral NaOH compared to acidic NaOH. The higher uncertainty of the Ar/170 wt losses compared to the other conditions is expected due to the non-adhering nature of an outer oxide layer.

That Ni 201 corroded more in neutral NaOH may be explained by the higher concentration of O^2−^ ions in molten NaOH at neutral conditions. Corrosion in NaOH is controlled by the OH^−^, which dissociates per equation (1), where H_2_O is the acidic component and O^2−^ is the basic component [20]. Thermal dissociation of NaOH occurs at high temperatures [21]. At high temperatures, the H_2_O generated by dissociation of the OH^−^ evaporates, which leads to a melt with an increase of O^2−^ compared to H_2_O. As per Le Chatelier's principle, adding H_2_O to the melt shifts the dissociation reaction (equation (1)) to the left and hence prevents formation of O^2−^ and will lower the basicity. While NiO is continuously forming (equation (8)), NiO may simultaneously react with O^2−^ to form NiO_2_^2−^ by equation (12) [9] at higher electrochemical potential.

From the dynamic polarization curves, it can be observed that the oxide formed on Ni 201 in Ar/p_H2O_(0.059) and Ar/p_H2O_(0.197) conditions seems to provide some passivation, which is interpreted from the rise in current at 0.9 V vs Na/Na + caused by the formation of oxide followed by a decrease in current density until 1.1 V vs Na/Na^+^. For Ni 201 in neutral NaOH, the current density is lower between 0.8 and 1 V vs Na/Na^+^ compared to the current density of the Ni 201 in acidic NaOH. This indicates that the formation of NiO is slower in neutral NaOH compared to acidic NaOH, as well as a decrease in current density was not seen for Ni 201 in neutral NaOH from 0.9 to 1.1 V vs Na/Na^+^, indicating that the oxide formed is less passivating. The reaction that increases current density levels at 1.15 V vs Na/Na^+^ for Ar/p_H2O_(0.059) and Ar/p_H2O_(0.197) conditions, starts at lower potentials when in neutral NaOH condition. The continuous increase in current from already 0.9 V vs Na/Na ^+^ could indicate that the reaction between O^2−^ and NiO occurs at lower potentials in neutral NaOH conditions which is in agreement with the significantly higher weight loss for neutral melt conditions compared to Ar/p_H2O_(0.059) and Ar/p_H2O_(0.197) conditions.

The oxide scales formed on the coupons during the static corrosion tests exhibit different morphologies. Specifically, the oxides formed in neutral NaOH display higher porosity and finer grain structures (as shown in Fig. 3). The smaller grains and porous microstructure may be a result of reaction between Ni and NiO with O^2−^ resulting in both NiO and Ni dissolution.

*The morphology of the oxide scales formed during the immersion tests indicate that Ar/*pH*2O(0.197) forms a denser and more protective oxide layer compared to the other two test conditions.* However, over time the oxides under such acidic conditions become more porous and form cracks. The growth of these oxides is non-parabolic, which may be due to their porous nature and the occurrence of cracks. The morphology and weight losses indicate that the oxide forming on Ni 201 in acidic NaOH may initially provide some passivation of the underlying Ni 201 and consequently slow down the corrosion as shown in Fig. 4A + B. However, over time the dense oxide appears fractured and with cracks and a layer of porous oxides form between the substrate and the outer dense oxides, Fig. 4C. NiO scales of duplex nature with an inner porous and an outer dense layer are frequently observed in literature [22,23]. Several formation mechanisms of the duplex oxide scales on nickel have been proposed. A review of the proposed mechanisms is provided by Kyung & Kim [24]. Some mechanisms attribute the stress induced by oxide growth as the primary factor leading to the initiation of porous scale formation, eventually progressing to a duplex scale [[25], [26], [27]]. Other theories emphasize the significance of the inward transport of oxygen through the initially developed single layer [28,15]. After spallation of the dense oxide scale on coupons exposed at Ar/p_H2O_(0.197) conditions, it may be followed by a crevice corrosion type of mechanism. The morphology of the inner porous layer (Fig. 4C) is similar to the oxides formed at Ar/170 and Ar/p_H2O_(0.059)/170 conditions, which could indicate a more basic local environment, resulting in the formation of a more porous and finer oxide.

The polarization curves show that passivation in dry argon shows higher current density levels than passivation obtained at Ar/p_H2O_(0.059) and Ar/p_H2O_(0.197) conditions. To obtain information of the passivation properties of oxides formed after longer durations it was attempted to carry out in situ Electrochemical Impedance Spectroscopy (EIS) over longer durations of exposure. However, the electrical contact between the coupon sample and the suspension wire was poor due to extensive corrosion of both. Due to the poor electrical contact, the data was not reproducible. For reliable and reproducible EIS measurements, it is crucial to ensure proper electrical contact between the sample and the potentiostat. This could be achieved by modifying the sample setup, for instance, by using rod samples, or by conducting EIS investigations ex situ after exposure.

## 5 Conclusion

The corrosion behavior of Ni 201 in NaOH at 600 °C under varying basicity was studied. Ni 201 corroded most in neutral NaOH. Ni 201 corroded less in NaOH kept under an atmosphere of argon with a partial pressure of water of 0.059 atm and even less under an atmosphere of argon with a partial pressure of water of 0.197 atm, which represents the most acidic condition in this study. Ni 201 developed oxide scales comprised of NiO under all test conditions with varying morphology. Coupons exposed to NaOH under atmospheres of argon with a partial pressure of water of 0.197 atm showed the densest oxide layer resulting in some level of passivation and less corrosion. However, Ni 201 did not form a fully protective oxide scales in NaOH even under such acidic conditions as the oxides showed non-parabolic growth over time and cracking after 340 h of exposure.

## Data availability statement

The data that has been used is confidential.

## CRediT authorship contribution statement

**Birgitte Stoffersen:** Writing – original draft, Visualization, Validation, Methodology, Investigation, Formal analysis, Conceptualization. **Daniel John Cooper:** Writing – review & editing, Supervision, Funding acquisition, Conceptualization. **Morten Stendahl Jellesen:** Writing – review & editing, Supervision, Project administration, Formal analysis, Conceptualization. **John Hald:** Writing – review & editing, Supervision, Project administration, Funding acquisition, Conceptualization.

## Declaration of competing interest

The authors declare the following financial interests/personal relationships which may be considered as potential competing interests:Birgitte stoffersen reports financial support was provided by Seaborg Technologies Aps. Birgitte stoffersen reports a relationship with Seaborg Technologies Aps that includes: employment. Daniel John Cooper reports a relationship with Seaborg Technologies Aps that includes: employment. If there are other authors, they declare that they have no known competing financial interests or personal relationships that could have appeared to influence the work reported in this paper.
